# Translating policy into practice: teacher agency amid cognitive and ecological constraints in enacting competency-based assessment

**DOI:** 10.3389/fpsyg.2026.1836710

**Published:** 2026-06-12

**Authors:** Fan Shi, Qing Yang, Ming-Xia Zhang, Xi-Ting Wang

**Affiliations:** School of Education Science, Qingdao University, Qingdao, China

**Keywords:** cognitive load, competency-based assessment, ecological constraints, epistemic beliefs, researcher-practitioner partnership, teacher agency

## Abstract

The global shift toward competency-based education places unprecedented demands on frontline teachers to enact dynamic, process-oriented assessments. However, translating these top-down policy mandates into daily practice within resource-constrained classrooms remains a significant challenge. To unpack this implementation gap, this study employed an exploratory, embedded single-case study through a researcher-practitioner partnership (RPP). In a 5th-grade STEM unit, university researchers provided a theoretical assessment scaffold based on the SOLO taxonomy, while the frontline teacher co-designed and enacted the context-specific rubrics, offering a crucial bottom-up reality check. Findings reveal that while these tools successfully made abstract competencies visible and supported responsive instruction, their continuous enactment faced severe real-world constraints. Structurally, immense data-processing demands collided with rigid instructional time, forcing the teacher to engage in rational triage, sacrificing assessment documentation to preserve core teaching. Culturally, the evidence-based system clashed with entrenched grading habits and a traditional culture of correctness, triggering student mistrust of peer evaluation. Our analysis indicates that these implementation gaps are primarily driven not by a deficit in teacher assessment literacy, but by the interplay of severe structural and cultural constraints. Without systemic interventions to alleviate these operational and epistemic burdens, complex assessment innovations will inevitably be reduced to superficial survival checklists. This study thus highlights the indispensable role of boundary-crossing RPPs not as a magic bullet to resolve structural issues, but as a critical reality check that renders the teacher’s invisible labor and the severe ecological constraints explicitly visible to policymakers.

## 1 Introduction

The global shift toward competency-based education has fundamentally altered the professional role of the teacher. Moving away from traditional knowledge transmission, modern educational paradigms increasingly demand that teachers act as sophisticated diagnosticians of student learning processes (NGSS Lead States, 2013; Osborne, 2013). This transition requires educators to integrate formative, process-oriented assessments into their daily instruction. However, bridging the gap between theoretical assessment frameworks and the practical realities of a teacher’s daily work remains a persistent global challenge. Tidemand and Tamborg (2025)’s systematic reviews identify deep-seated systemic contradictions that hinder this transition, where ambitious reform goals frequently clash with the inherent constraints of established school organizations. Furthermore, even under reform-oriented curricula, teacher’ practices often remain product-oriented, prioritizing the acquisition of static content knowledge over the complex, dynamic processes of competency development (Nielsen and Nielsen, 2021).

In China, this challenge is acutely evident within the context of the key competencies-based curriculum reform (locally known as *Hexin Suyang*) (Ministry of Education, P. R. China, 2022). To support this reform, a recent national policy mandates “Comprehensive Assessment” (*Xueye Shuping*), which requires teachers to conduct nuanced, individualized evaluations of each student’s developmental process (Central Committee of the Communist Party of China and the State Council, 2020). Despite the clarity of this top-down policy directive, frontline science teachers consistently prioritize tasks with lower cognitive demands (e.g., conceptual recall) while neglecting more complex competencies like scientific thinking or deep interpretation of data (Pérez de Landazábal et al., 2012). Moreover, they face a profound practical dilemma: they are expected to evaluate these complex competencies but must do so within ecologies constrained by large class sizes, rigid instructional time, and a severe lack of operational assessment tools (Liu and Wang, 2023; Zhan et al., 2021).

Concurrently, the dominant discourse in educational research often attributes the failure of such reforms to an individual professional deficit namely, a lack of teacher assessment literacy (Hung and Wu, 2024; Xu and Brown, 2016). While assessment tools continue to proliferate under idealized conditions, this individualistic deficit model frequently overlooks the micro-level structural and cultural constraints that severely restrict a teacher’s capacity to enact these tools in real-world practice. Following the ecological perspective (Priestley et al., 2013, 2015), implementing complex educational innovations is not merely a matter of inherent individual capacity, but a situated achievement that depends heavily on available material scaffolds and a supportive working ecology. In standard public school settings, the sheer volume of structural constraints such as rigid 40-min lesson periods and large class sizes makes it highly unrealistic to rely solely on individual frontline teachers to translate top-down assessment policies into dynamic classroom practices. Consequently, there is an urgent need for boundary-crossing collaboration to share this immense cognitive and operational burden, yet there remains a significant gap in understanding the actual practical frictions teachers encounter when enacting these high-fidelity assessments.

To address this, and explore how teachers navigate the translation of competency-based assessment policy into classroom reality, this study employs a Researcher-Practitioner Partnership (RPP) (Chen et al., 2022). By collaborating with a frontline teacher to co-design and field-test a structured assessment system in a specific STEM instructional unit (“Making a Thermos Flask”) in a Grade 5 science classroom, this study seeks to unpack both the pedagogical affordances and the practical constraints of this process. Instead of evaluating the assessment tools in isolated, controlled conditions, we embed our investigation within the intense, daily instructional flow of a standard public school classroom. By exploring the structural and cultural constraints in a real-world setting, this study aims to reveal how these ecological pressures translate into specific cognitive and epistemic burdens for both teachers and students.

Specifically, the study is guided by two research questions:

*RQ1*: How does the competency-based assessment framework support students’ learning and teacher’s instructional adjustments during the STEM unit?

*RQ2*: What structural and cultural constraints emerge when the teacher enacts this assessment framework in a standard, resource-constrained primary classroom?

## 2 Literature review and theoretical framework

### 2.1 The theoretical architecture of comprehensive assessment (Xueye Shuping)

**Figure 1:**
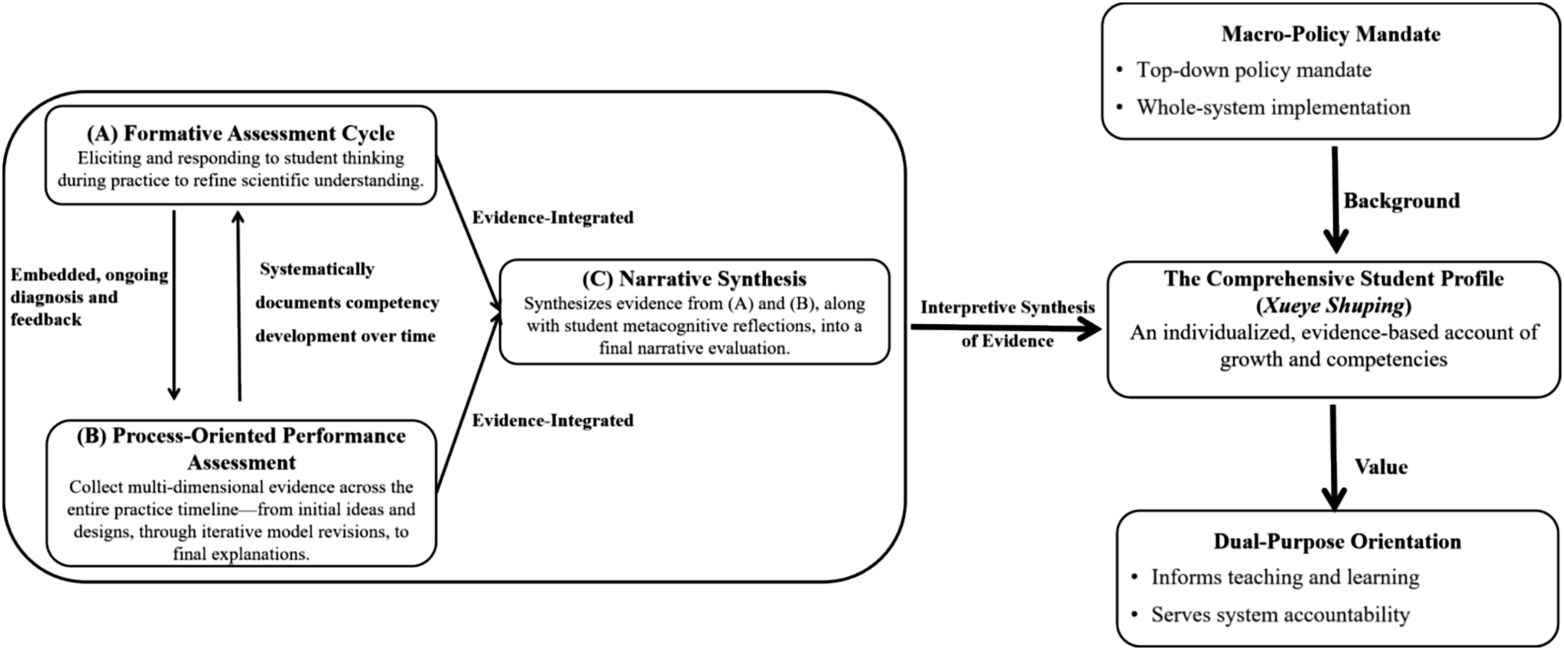
Theoretical conceptualization of China’s comprehensive assessment as a synthesized framework.

As science education shifts from content delivery to competency development, assessment frameworks must evolve accordingly. In China, this shift is operationalized through the national mandate for comprehensive assessment. This framework prioritizes the process-oriented nature of student development over static scores, requiring that assessment reflects the development of key competencies. This evaluative shift is operationally defined by three core characteristics: comprehensive, process-oriented, and performance-based, as conceptualized in Figure 1. Together, these characteristics intend to move assessment beyond a reductionist score toward a thick description of student growth (Clandinin, 2004; Heritage, 2010; National Research Council, 2014; Ruiz-Primo and Furtak, 2007; Wiggins, 1990). However, implementing this high-fidelity framework in high-pressure classrooms remains a significant operational challenge, as teachers often lack the technical tools to translate messy process observations into actionable feedback (Liu and Wang, 2023).

### 2.2 Methodological scaffold: using the SOLO taxonomy for assessment design

To operationalize the abstract goals of China’s competency-based assessment policy into manageable classroom practices, teachers require a clear metric to categorize the complexity of student performance. In this study, we utilize the SOLO taxonomy (Structure of the Observed Learning Outcome) (Biggs and Collis, 1982) as a methodological scaffold to design our assessment rubrics. Unlike Bloom’s taxonomy, which often fragments knowledge into discrete cognitive operations, SOLO is hierarchical and structural, focusing on the complexity and integration of student responses rather than just the quantity of correct facts. The taxonomy categorizes understanding into five progressive levels, which makes it uniquely suitable for the process-oriented demands of scientific assessment, as it allows for the differentiation between surface-level performance and deep conceptual understanding. Recent empirical scholarship in science education supports the use of SOLO for assessing dynamic practices. Studies have demonstrated its efficacy in mapping student progressions in complex tasks such as scientific argumentation and inquiry-based modeling (Chin et al., 2022). For instance, in assessing argumentation, SOLO allows teachers to distinguish between students who merely list data (Multi-structural) and those who use data to justify a claim and rebut counter-arguments (Relational).

Rather than testing the taxonomy itself which is well-established, this study uses SOLO as a practical tool to provide the granular grading logic necessary to operationalize the comprehensive assessment policy, converting abstract educational goals into a concrete, progressive rubric for student self-assessment, peer assessment, and teacher evaluation.

### 2.3 Theoretical lens: an ecological perspective on assessment implementation

Implementing a competency-based assessment system is not merely a matter of a teacher’s individual skill or assessment literacy. It is profoundly shaped by the classroom environment. To systematically analyze the challenges of enacting these assessments, this study adopts the ecological perspective on teacher agency (Priestley et al., 2013, 2015). In this widely recognized educational framework, teacher agency is not viewed as a fixed personal capacity, but rather as an emergent phenomenon that is achieved through the interaction between the teacher and their specific working ecology. Priestley et al. (2013, 2015) argue that a teacher’s ability to implement pedagogical innovations is enabled or constrained by different dimensions of their environment, primarily the material resources available, the structural conditions of the school, and the underlying cultural beliefs of the actors involved.

**Figure 2:**
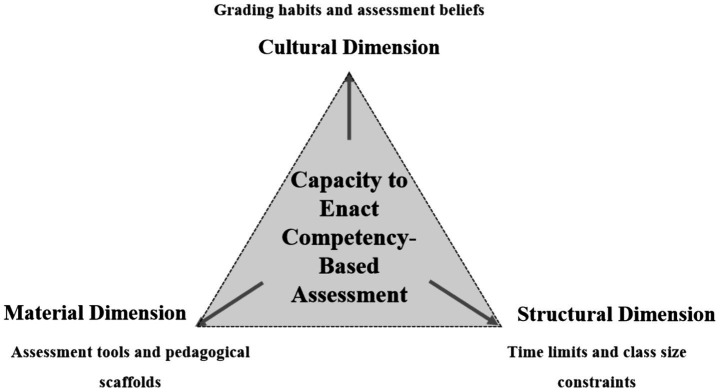
The analytical lens of ecological perspective.

In the context of our study, this ecological framework provides a highly practical analytical lens (Figure 2). Rather than merely judging the assessment tool’s inherent validity, we systematically examine how the newly introduced material dimension (the SOLO-scaffolded rubrics) interacts with standard structural conditions (e.g., instructional time limits, class sizes) and deeply entrenched cultural beliefs (e.g., existing grading habits). This continuous interplay ultimately shapes the teacher’s practical capacity to enact competency-based assessment.

By applying this ecological lens, we aim to move beyond simply judging the assessment tool as a success or failure. Instead, we systematically unpack the specific structural and cultural frictions the practitioner encountered when attempting to enact the comprehensive assessment policy in a standard primary science classroom.

## 3 Methodology

### 3.1 Overall research design: an exploratory embedded single-case study

This study adopts an exploratory, embedded single-case study design (Yin, 2017) facilitated through a Researcher-Practitioner Partnership (RPP). As highlighted in recent literature within the Chinese educational context, bridging the gap between top-down comprehensive assessment policy and bottom-up classroom reality requires boundary-crossing collaboration (Chen et al., 2022; Fang et al., 2022). In this study, the external university researchers (first and second authors) collaborated with a frontline primary science teacher (third author). To navigate the complex translation from policy to practice, this partnership adopted a synergistic, co-design approach rather than a rigid division of labor. Initially, the university researchers introduced the SOLO taxonomy and elucidated its alignment with core scientific competencies, guiding the practitioner in conceptualizing the macro-theoretical framework of comprehensive assessment (as detailed in Tables 1, 2). Building upon this shared theoretical foundation, the researchers and the practitioner iteratively negotiated and co-developed the unit-specific assessment tools (e.g., the student self-assessment scales and peer-assessment rubrics). During the subsequent instructional phase, the practitioner led the pedagogical enactment to test these tools against classroom realities, while the university researchers conducted continuous on-site observation and systematic data analysis. We selected this collaborative approach to explore how a competency-based assessment system functions within the authentic constraints of a high-stakes classroom ecology.

**Table 1:** The multidimensional evaluative framework and assessor matrix.

Primary dimension	Secondary indicator	Description	Primary assessor(s)
Scientific concept	Specific scientific concepts	Ability to comprehend key concepts within scientific disciplines (e.g., conservation of energy, force interactions) and interdisciplinary concepts (e.g., structure and function, systems and models).	Teacher
	Nature of science	Ability to recognize the characteristics inherent in scientific knowledge and its development process (e.g., the testability and evolving nature of science).	Student
Scientific thinking	Modeling	Ability to abstract and generalize from empirical facts or phenomena, demonstrating initial competence in model understanding, construction, evaluation and revision.	Teacher & student
	Reasoning & argumentation	Ability to apply analytical, comparative, and deductive thinking to establish relationships between evidence and explanations and propose justified reasoning.	Teacher & student
	Creative thinking	Ability to analyze and consider problems from different perspectives, thereby proposing novel and valuable viewpoints and solutions.	Teacher & student
Scientific practices	Scientific inquiry	Competence demonstrated in inquiry activities, including: posing questions, formulating hypotheses, planning and conducting investigations, and communicating and analyzing results to draw conclusions.	Student & peer
	Technological & engineering practice	Competence demonstrated in technological/engineering activities, including: encompassing understanding of processes, using tools/materials to implement plans, iterating designs based on outcomes, and creating models to test or demonstrate principles.	Student & peer
	Self-directed learning	Ability to proactively employ cognitive and non-cognitive strategies, including goal-setting, strategy selection, process monitoring, and reflection.	Student
	Collaborative learning	Competence in communication, organization, and coordination demonstrated during group problem-solving.	Peer
Attitude & responsibility	Learning interest	Curiosity and enthusiasm for scientific phenomena and learning.	Student
	Scientific attitude	A stable psychological inclination toward science, including respect for evidence, questioning, and critical thinking.	Student
	Social responsibility	A sense of responsibility for environmental protection, resource conservation, and sustainable development, and an awareness of the role of science in society.	Student

**Table 2:** Level descriptors for the ‘reasoning & argumentation’ dimension.

Secondary indicator	Level description
Reasoning & argumentation	L1: Can state a claim but provides no valid evidence or reasoning, failing to demonstrate a complete reasoning process. L2: Support a claim by describing personal experience, prior knowledge, or specific experimental results, constituting a data-based argument. L3: Analyze available data, selects valid evidence to support a claim, and explains the connection between the claim and evidence within a specific context. L4: Use inductive generalization or deductive abstract relationships to support a claim, articulating more universal relationships, principles, or laws, thereby demonstrating a complete argumentation process.

The case is defined as the enactment of the assessment system within a specific instructional unit (Making a Thermos Flask) in a Grade 5 science classroom. To ensure analytical depth and triangulate findings, we established three embedded units of analysis: (a) the assessment tools, evaluating the validity and feasibility of the SOLO-scaffolded instruments designed for the unit; (b) student learning trajectories, examining both macro-patterns of class performance and micro-processes of focal students’ metacognitive development; (c) the ecological constraints, analyzing the structural costs and epistemic friction that emerged during the system’s enactment.

### 3.2 Research context and participants

The study was conducted in a public primary school in Qingdao, China, a typical setting representing the standard urban school ecology in China (e.g., large class sizes of 33 students, rigid 40-min lesson periods). The participants were intact Grade 5 students (aged 10–11) who had basic experience with inquiry-based science but lacked exposure to systematic process-oriented assessment. The practitioner-researcher (the third author), with 3 years of experience, facilitated the instructional unit. The teacher’s involvement provided valuable insider (emic) access for both the enactment of the curriculum and the interpretation of data.

To gain in-depth qualitative insights, this study also employed a purposive sampling strategy to select four focal students (StA-StD). These students were selected not as representatives of a single proficiency level, but to capture a spectrum of distinct performance profiles observed within the multi-dimensional evaluation framework (see Supplementary Appendix 1). They are referred to by neutral identifiers (StA-StD) throughout this report to ensure anonymity and clarity.

The instructional context was a STEM-integrated engineering unit titled “Making a Thermos Flask” (4 class periods). This unit was selected because it requires the integration of disciplinary knowledge (heat transfer), engineering practices (design and iteration), and social responsibility (cost and material analysis), providing a rich context for assessing multidimensional key competencies.

### 3.3 The competency-based assessment system

The assessment system was designed to operationalize the abstract “key competencies” defined in Ministry of Education, P. R. China, (2022) into observable classroom practices. The development and enactment of this system followed a structured trajectory, from framework construction, to tool development, and finally to instructional embedding.

#### 3.3.1 Framework construction: operationalizing competencies via SOLO

To transform abstract curriculum standards into measurable classroom indicators, we developed the evaluative framework through a three-step process: (1) extracting secondary indicators (e.g., Modeling, Argumentation) from a content analysis of national standards to form a multi-dimensional matrix (Table 1); (2) mapping these indicators onto four progressive proficiency levels (L1–L4) using the SOLO taxonomy as a methodological scaffold (Table 2); and (3) verifying content validity through two rounds of Delphi consultation with eight science education experts. This process ensured that the resulting level descriptors (Supplementary Appendix 2) were both scientifically rigorous and developmentally appropriate for Grade 5 students.

#### 3.3.2 Development of multi-source assessment tools

Based on the validated framework, we collaborated to develop a suite of context-specific assessment tools (see Supplementary Appendices 3–5). *Student Self-assessment Scales* used student-friendly language to scaffold metacognitive reflection on scientific practices. *Peer-assessment Rubrics* focused on observable criteria anchored in scientific principles (e.g., whether a peer’s device was constructed ‘based on scientific principles of heat’). Finally, the *Teacher’s Assessment Portfolio* combined a *Reflective Observation Log which* designed to document both student competencies, and the systemic structural/cultural frictions encountered during enactment with an *Energy Concept Test* (adapted from TIMSS, Mullis and Martin, 2017) to measure conceptual attainment. This multi-source approach ensured a balanced evaluation of both higher-order procedural competencies and foundational knowledge.

#### 3.3.3 Instructional enactment and assessment nodes

**Figure 3:**
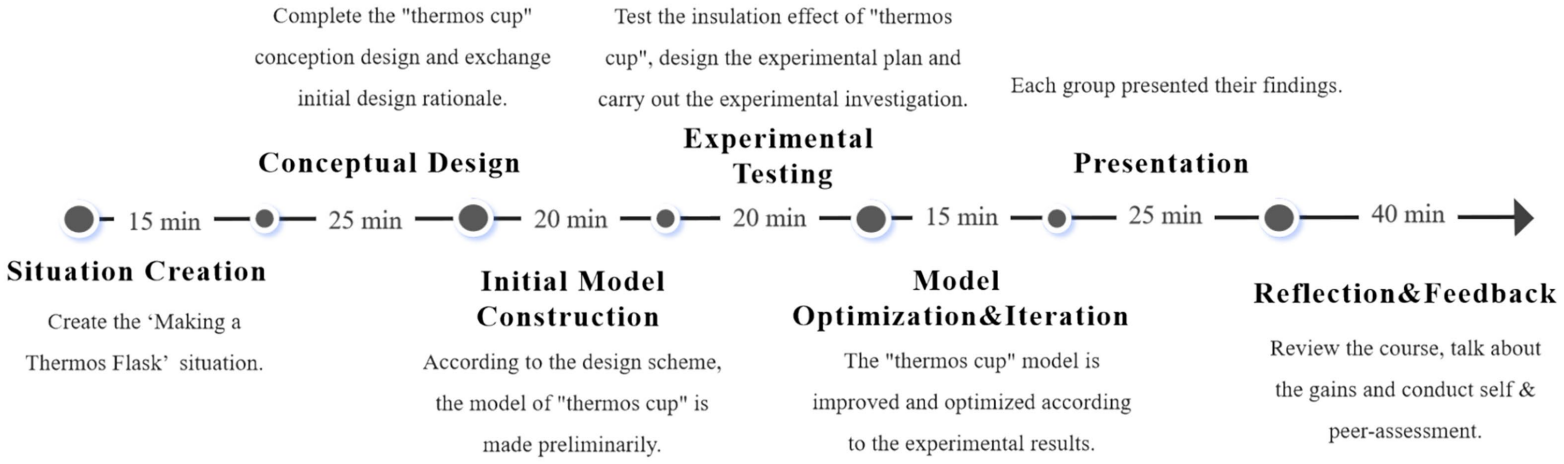
Instructional flow and timeline of the “Making a Thermos Flask” engineering unit. The comprehensive assessment system was embedded at key nodes within this process (see Table 3 for details).

The competency-based assessment system was strategically embedded into the instructional fabric of the STEM-integrated unit, Making a Thermos Flask (four class periods). As illustrated in Figure 3, the instructional flow followed a project-based logic. To ensure that assessment served a formative function, specific evaluation activities were positioned at critical instructional nodes within this flow. This embedded arrangement ensured that students engaged in metacognitive reflection precisely when interpreting their data, thereby transforming the assessment from a retrospective summary into a dynamic scaffold for subsequent model iterations.

### 3.4 Data collection

The research team employed a convergent mixed-methods strategy to capture the dynamic enactment of the assessment system. As detailed in Table 3, data collection was systematically aligned with the four instructional nodes, ensuring a comprehensive evaluation of both student learning trajectories and ecological constraints. During the enactment phase (Nodes 1–3), we prioritized capturing the real-time formation of competencies through *Student Self-Assessment Scales*, the teacher’s *Reflective Observation Log*, and opportunistic interviews with focal students.

**Table 3:** Data collection strategy aligned with instructional enactment nodes.

Case phase & instructional node (from Figure 3)	Assessment focus & embedded tools (with specific items in Supplementary Appendices 3–5)	Primary function & data collection purpose
• Teacher preparation	• Teacher semi-structured interview	To establish baseline for pedagogical plans, expectations, and pre-existing assessment beliefs.
• After conception design/initial model construction (Node 1)	• Student Self-assessment Scales: Q5 (creative thinking), q8 (engineering practice) • Teacher’s reflective observation log: focus on evidence for *Creative Thinking* and *Engineering Practice* • Focal student opportunistic interviews: on initial design rationale.	To diagnose students’ initial conceptualization and meta-cognitive awareness of scientific practices. To capture early reasoning processes and document initial structural constraints (e.g., time management).
• After experimental testing (Node 2)	• Student Self-assessment Scales: Q4 (reasoning & argumentation), Q7 (scientific inquiry) • Teacher’s reflective observation log: focus on evidence for *reasoning & argumentation*. • Focal student opportunistic interviews: on interpreting data and forming arguments.	To diagnose student’ ability to transform data into evidence. To trigger instructional adjustments and record sociotechnical frictions during testing To evaluate group inquiry dynamics.
• After model optimization/iteration (Node 3)	• Student Self-assessment Scales: Q3 (modeling), Q6 (self-directed learning), Q10 (attitude); • Teacher’s reflective observation log: focus on evidence for *iterative modeling* and *collaborative problem-solving*. • Focal student opportunistic interviews: on models optimization and iteration.	To assess capacity for iterative improvement based on feedback and group collaboration efficacy. To validate adjustments and identify persistent ecological barriers.
• After presentation/reflection & feedback (Node 4)	• Student Self-assessment Scales: Q1, Q2 (NOS); Q9 (interest); Q11 (attitude), Q12 (responsibility) • Peer-assessment rubric: all items (completed for final, comprehensive evaluation) • Energy concept survey (Supplementary Appendix 5B) • Student reception questionnaire • Focal student & teacher semi-structured interviews: in-depth, retrospective	To synthesize a comprehensive data set of the entire learning journey. To evaluate final conceptual understanding, system reception, and holistic impact. To generate the final comprehensive student competency profile

In the summative phase (Node 4), we evaluated holistic outcomes through *Peer-Assessment Rubrics*, the *Energy Concept Test*, and a *Student Reception Questionnaire* (*N* = 32 valid responses; Cronbach’s *α* = 0.702). This reliability value meets the acceptable threshold for exploratory research. To triangulate these findings, in-depth retrospective interviews were conducted with the practitioner to reconstruct implementation barriers and with focal students to probe their learning experiences.

### 3.5 Data analysis

A convergent mixed-methods strategy was employed to analyze the collected data, aiming to integrate macro-patterns with micro-mechanisms. Quantitative data from the *Energy Concept Test* and *Student Reception Questionnaire* were analyzed using descriptive statistics solely to identify general trends in learning outcomes and system acceptance. Given the exploratory nature of this single-case study, these quantitative elements served as a descriptive backdrop for triangulation rather than for causal or comparative claims; inferential statistics were omitted as the sample represents a single, intact classroom.

Accordingly, the primary analytical weight was placed on the qualitative dataset, which comprised semi-structured interviews, the teacher’s *Reflective Observation Logs*, and open-ended assessment artifacts. The qualitative analysis followed an iterative thematic approach (Braun and Clarke, 2006), progressing systematically from inductive grounding to deductive theoretical integration. Initially, researchers independently engaged in line-by-line reading to generate data-driven codes, such as specific classroom frictions or student hesitations. These were subsequently clustered into broader descriptive themes before being mapped against the three dimensions of the ecological perspective (Priestley et al., 2015): material, structural, and cultural. This trajectory, illustrated in Table 4, allowed us to elevate descriptive patterns into an explanatory theoretical framework.

**Table 4:** Exemplar of the data analysis trajectory: triangulating multi-source data to theoretical dimensions.

Analytical phase / Theme development	Representative data Interpretation
Data sources & representative segments	•Student interview (StB): “I do not agree with the score some peers gave me; it feels unfair. They might not have seen all the work I did.” •Student interview (StA): “I trust the teacher’s opinion the most. She knows the right answer.” •Quantitative pattern (survey): While general acceptance was high, a notable minority (12.5%) expressed specific reservations about the fairness of peer assessment (Item 6). • Teacher’s reflective log: Why is it so hard to stop looking for the “correct answer”?
Step 1: Inductive coding	• Mistrust of peer grading • Preference for teacher’s authoritative judgment • Fairness defined as getting the correct answer • Teacher’s internal struggle with traditional grading habits
Step 2: Descriptive theme	Entrenched beliefs about assessment: Both students and the teacher experience discomfort and resistance when transitioning from a traditional, teacher-centered grading system to a distributed, evidence-based peer assessment system.
Step 3: Theoretical mapping	Cultural dimension The data reveal a profound cultural friction. The new assessment rubrics demand a culture of evidence, which collides with the entrenched culture of correctness and traditional authority structures existing within the classroom’s ecology.

### 3.6 Researcher positionality and validity protocols

To leverage the researcher–practitioner Partnership (RPP) while ensuring analytical rigor, we established a clear division of labor. The external researchers (first and second authors) conducted the primary interpretive analysis via analytical triangulation, engaging in collaborative coding until a full consensus was reached on all qualitative data. Concurrently, the practitioner-researcher (third author) facilitated enactment, generated raw data, and provided factual member checking to verify specific classroom events. This boundary-crossing collaboration (Chen et al., 2022) explicitly confined the practitioner to factual verification, ensuring the external researchers’ interpretations of ecological constraints remained objective and uncompromised. Furthermore, to monitor potential interpretive bias from the practitioner-researcher, the external researchers deliberately sought alternative interpretations. A prominent example of this disconfirming case analysis occurred regarding the incomplete assessment logs. Initially, the practitioner-researcher interpreted her real-time documentation failure as a personal deficit, framing it as a lack of implementation fidelity. However, by cross-referencing reflective logs with observational data, the external researchers challenged this bias, proposing an alternative structural interpretation: the incomplete documentation was not a personal failure, but a rational triage (Lipsky, 2010)—a necessary protective decision forced by severe ecological constraints. This collaborative friction ensured that final interpretations were elevated from individual self-reflection to systemic ecological critique.

## 4 Findings

Applying the ecological perspective as our analytical lens, we present the findings in two main sections. First, we examine the material dimension, detailing how the assessment rubrics successfully supported students’ learning and the teacher’s instruction. Second, we unpack the practical challenges encountered during implementation, specifically the structural and cultural constraints that hindered the smooth enactment of the assessment system.

### 4.1 The material dimension: scaffolding learning and instruction

The competency-based assessment rubrics, scaffolded by the SOLO taxonomy, functioned as effective material tools in the classroom. They provided a tangible framework that made abstract scientific competencies visible to both students and the teacher.

#### 4.1.1 The rubric as a cognitive scaffold: visualizing expectations for students

In the ecological framework, the SOLO-scaffolded rubrics functioned as a crucial material tool. They translated abstract scientific competencies into a shared, visible discourse that students could understand. Survey data (Table 5) provided a macro-level view of this adaptation. While most students agreed the rubrics raised competency awareness (Item 10, 93.8%) and clarified learning paths (Item 11, 96.9%), agreement was slightly lower (Item 9, 84.4%) regarding the system’s facilitation of independent self-correction. This discrepancy reflects the authentic classroom reality: visualizing the gap between current performance and expected standards is easier than fully internalizing these criteria to independently regulate learning.

**Table 5:** Summary of student reception to the competency-based assessment system (*N* = 32).

Dimension/key item focus	% Positive (agree + strongly agree)	Key insight
Student adaptation		Students demonstrated high readiness for and acceptance of the new assessment method.
• Willingness to use assessment rubrics (Item 1)	96.9%	
• Overcoming fear of score-based evaluation (Item 5)	100.0%	
Perceived accuracy		The system was largely seen as a fair and comprehensive reflection of their competencies.
• Fair reflection of academic performance (Item 7)	93.8%	
• Comprehensive assessment of core competencies (Item 8)	93.8%	
Impact on learning & capacity development		The system was perceived as effective in raising awareness and guiding learning, though the depth of self-regulatory application varied.
• Facilitating self-reflection and correction (Item 9)	84.4%	
• Raising awareness of core competencies (Item 10)	93.8%	
• Clarifying future learning paths (Item 11)	96.9%	
Notable tension		A minority of students questioned the fairness of peer assessment, highlighting a cultural challenge.
• Agreement with evaluations from peers/teacher (Item 6)	87.5%	

Qualitative data triangulates this survey finding and provides deeper insight into this scaffolding mechanism. Following the *Model Optimization & Interaction* (Instructional Node 3, Table 3) phase, focal student StD explained how the rubric guided him: “Reading the rubric... I saw that options C and D were the better levels. This lets me know that in the future, I should work towards the direction described in C and D.” StD’s use of the phrase “in the future” perfectly aligns with the discrepancy observed in Item 9, where the rubric effectively functioned as a roadmap for goal-setting, even if the student had not yet fully mastered the ability to reach that goal independently.

Furthermore, the practitioner-researcher’s retrospective interview confirmed that the rubrics materially altered the classroom interactions. She noted: “The most tangible change was hearing students use the language from the rubrics in their group discussions... e.g., ‘I think our reasoning is at level 3 because we compared our data...’” This observation demonstrates that the assessment tool did not just sit on paper, and it successfully penetrated the daily classroom discourse. By externalizing abstract scientific standards into concrete descriptors, the tool scaffolded the students’ emerging awareness, helping them transition from passively completing tasks to actively discussing the quality of their scientific practices.

#### 4.1.2 The rubric as a diagnostic tool: enabling responsive instruction

For the teacher, the SOLO-scaffolded rubrics served as a critical material diagnostic tool that triggered immediate instructional adjustments. A clear example occurred after the *Experimental Testing* phase (Instructional Node 2), when the teacher collected the students’ self-assessment scales regarding *Reasoning & Argumentation*. The initial data revealed a significant gap in student understanding, which the practitioner-researcher documented in her *Reflective Observation Log*:

*“Collected self-assessments.*

*Worrying sign. A cluster of students are hesitant, asking which one is me?.*

*The bridge is missing.”*

Opportunistic interviews confirmed that while students had successfully performed the physical task (comparing water temperatures), they could not connect these actions to the abstract criteria of scientific argumentation on the rubric. Focal student StB articulated this struggle clearly: “I got stuck. I read the options, but none of them matched what I actually did... I didn’t know which box to tick.” Faced with this data-driven diagnosis, the teacher enacted a responsive pedagogy rather than pushing forward with the pre-planned schedule. She explicitly deconstructed the logic of argumentation by contrasting the temperature data from two different student groups, modeling how to transform a raw observation (*‘Ours kept the water warmer’*) into an evidence-based claim aligned with the Level 3 descriptor.

The impact of this targeted instruction was confirmed by student reflections. Focal student StC described his moment of realization: “The teacher showed us the data from Group A and B. Suddenly I got it. It’s not just about saying ‘ours is hot’, you have to use the numbers to prove why.” Concurrently, the process of diagnosing student thinking catalyzed a shift in the teacher’s own instructional focus. Reflecting on the enactment, she articulated a profound change in her values: “I began to see that a ‘messy’ process with strong reasoning was more valuable than a clean result achieved by luck or imitation. Assessing the ‘how’ and ‘why’ became my new focus.” In this instance, the material tool (the rubric) did more than just measure learning; it actively reshaped the instructional process. It forced the teacher to confront the reality of student thinking and provided the necessary feedback to bridge the gap between doing a science activity and understanding scientific reasoning.

### 4.2 The implementation challenges: structural and cultural constraints

Despite the supportive role of the assessment rubrics in scaffolding learning and instruction, enacting this formative system within a standard public school ecology presented severe practical challenges. The collaborative analysis revealed that the practitioner faced significant structural and cultural constraints, which ultimately forced her to make compromises in how the assessment was implemented.

#### 4.2.1 The structural constraint: cognitive overload and time rigidity

The most prominent implementation gap arose from the conflict between the demanding nature of process-oriented assessment and the rigid structural limits of the school ecology. This structural constraint manifested at both the micro-level of classroom instruction and the macro-level of institutional workload.

First, within the classroom, the practitioner faced a severe time-documentation conflict. Tracking and documenting the dynamic learning trajectories of 33 students within a standard 40-min instructional block proved to be an overwhelming task. The practitioner-researcher’s reflective log poignantly captured this real-time tension:

*“Great! Group 3 is deep in a debate about insulation layers;*

*I wanted to record StA’s brilliant argument on the rubric. But I had to rush to Group 4 for a safety check with the hot water.*

*When I came back, the moment was gone.”*

Faced with the physical impossibility of providing immediate pedagogical guidance while simultaneously completing detailed assessment documentation, the teacher engaged in what Lipsky (2010) describes as rational triage. She consistently prioritized the immediate safety and instructional needs of the students (teaching) over the bureaucratic demand of filling out the assessment logs (documenting).

Second, beyond the classroom, the assessment reform imposed a massive burden of invisible labor due to a lack of institutional infrastructure. While the policy mandated comprehensive evaluation, it provided no standardized, operational tools. In our RPP, while the university researchers provided the theoretical scaffold (the SOLO taxonomy), translating these abstract frameworks into context-specific rubrics still required immense practical labor from the teacher. The practitioner noted in her retrospective interview: “[Even with the research team’s theoretical support]*, building the specific rubrics for my lessons was a massive undertaking... This intensive labor is invisible in our workload calculations. Without formal time allocation, it feels like a voluntary ‘add-on’.”* This reveals that the existing institutional structure fails to recognize assessment design and data analysis as official, billable professional work, making the sustained enactment of such high-fidelity assessments highly vulnerable.

#### 4.2.2 The cultural constraints: epistemic beliefs and assessment habits

Beyond physical and structural limitations, implementation encountered deep-seated cultural resistance from both students and teachers. According to Priestley et al. (2015), cultural constraints in an ecological framework refer to the deeply held beliefs and values that shape classroom behaviour. The new assessment rubrics demanded a culture of evidence, in which processes are evaluated and claims are justified. This clashed directly with the culture of correctness that is prevalent in traditional, exam-oriented environments.

For students, this cultural friction manifested as noticeable discomfort with peer assessment. Questionnaire data in Table 5 showed although the majority of students (93.8%) agreed that the assessment fairly reflected their academic performance (Item 7), a significant minority (12.5%) expressed concerns about the fairness of peer grading (Item 6). This contrast highlights that the core tension was not about the rubric’s general fairness, but specifically about the redistribution of evaluative authority. As illustrated in the data analysis trajectory (see Table 4), qualitative interviews exposed the root of this mistrust. StB complained, “I don’t agree with the score some peers gave me. It feels unfair. They might not have seen all the work I did.” Similarly, StA reflected a strong reliance on traditional teacher authority: “I trust the teacher’s opinion the most. She knows the right answer.” These responses indicate that students, habituated to a classroom ecology where evaluative authority resides solely with the teacher, struggled to accept peers as valid evaluators.

Crucially, the practitioner-researcher also experienced this cultural constraint internally. Shifting from a traditional grader to a facilitator of scientific practice required her to constantly fight against her own professional habits. Her reflective log offered a candid confession of this struggle:

*“[During the model testing] Caught myself again today.*

*Instinctively praised Group 2 just because their model looked ‘neat’.*

*Almost ignored Group 5 because their design failed, even though their hypothesis about air pockets was brilliant.*

*Why is it so hard to stop looking for the ‘correct answer’?”*

This raw reflection highlights that enacting competency-based assessment is not merely a technical skill to be learned. It requires a profound, and often difficult, cultural shift in how both teachers and students perceive the nature of learning and evaluation.

## 5 Discussion, conclusion and implications

### 5.1 Discussion

This study set out to explore the practical implementation of a competency-based assessment system through a researcher–practitioner partnership (RPP) in a primary science classroom. In this collaborative framework, while the university researchers introduced the top-down theoretical scaffold (the SOLO taxonomy), it was the frontline practitioner who provided a crucial, bottom-up reality check. Viewed through the ecological perspective on teacher agency (Priestley et al., 2015), our findings offer critical insights into why theoretically sound assessment policies often falter in frontline classrooms.

#### 5.1.1 Moving beyond the assessment literacy deficit: the necessity of boundary-crossing collaboration

For decades, the dominant discourse in educational assessment has heavily centered on teacher assessment literacy as the primary determinant of successful assessment reform (Abell and Siegel, 2011; Xu and Brown, 2016). Within this paradigm, when innovative formative assessments fail to become embedded in classrooms, the cause is often attributed to a professional deficit. Namely, that teachers lack the requisite skills or knowledge to implement complex practices (Hung and Wu, 2024; Mertler, 2004). However, the qualitative evidence from this study challenges the sufficiency of this individual-deficit model. In our case, the practitioner deeply understood the theoretical framework and actively co-designed the context-specific rubrics. She did not lack assessment literacy, rather, her implementation was severely restricted by the structural constraints of the classroom ecology. As demonstrated in our findings, tracking the dynamic, iterative scientific practice of 33 students within a rigid 40-min lesson created an immense structural and temporal load that simply exceeded the carrying capacity of a single teacher. We argue that a well-designed, theoretically rigorous assessment tool cannot function independently of its environment. Even highly assessment-literate teachers will struggle to sustain these practices if the structural dimensions of their work environment remain fundamentally incompatible with the demands of process-oriented evaluation.

Consequently, bridging this implementation gap requires moving beyond merely training individual teachers, toward fostering boundary-crossing collaboration (Chen et al., 2022). In this Researcher-Practitioner Partnership (RPP), the university researchers absorbed the heavy cognitive and theoretical labor of translating abstract policies into the SOLO-scaffolded rubrics. Conversely, the frontline teacher provided an indispensable reality check. By embedding these tools into authentic instructional nodes, she revealed the exact points where theoretical ideals collided with ecological limits. This synergistic process underscores that enacting competency-based assessment in public schools is not a solo endeavor of teacher literacy, but a collaborative achievement of ecological adaptation.

#### 5.1.2 Rational triage as a coping mechanism for cognitive overload

When the idealized demands of the assessment system collided with the physical constraints of the classroom, the teacher engaged in rational triage (Lipsky, 2010). Rather than perceiving the incomplete assessment logs as a mere lack of implementation fidelity, our ecological perspective unveils this as a profoundly protective professional decision. In order to ensure the effective fulfilment of core pedagogical tasks, such as the maintenance of classroom safety, the troubleshooting of experimental setups, and the guidance of dynamic student discussions, the teacher was compelled to relinquish the administrative burden of completing the rubrics in real-time. This classroom-level reality carries a critical implication for systemic assessment reform. It is evident that, in the absence of structural interventions such as the reduction of class sizes, the provision of institutional buffer time, or the employment of technological offloading (for example, the utilization of AI-assisted tools to alleviate the data-processing load), the introduction of complex formative assessments will inevitably result in teachers being forced into a triage model, thereby reducing high-fidelity tools to sporadic checklists.

#### 5.1.3 The deep roots of epistemic friction

Furthermore, our findings highlight that assessment tools cannot automatically overwrite entrenched classroom cultures. The resistance to peer assessment from students, and the teacher’s internal struggle to move away from seeking the correct answer, underscore that competency-based assessment requires a profound paradigm shift. Moving from a traditional culture of correctness to a culture of evidence is a slow, socially negotiated process (Driver et al., 1994). These deep-seated epistemic beliefs act as powerful cultural constraints within the classroom ecology. As this study suggests, such cultural shifts require sustained, collaborative reflection and transitional buffer zones, far beyond the mere introduction of a new rubric.

### 5.2 Conclusion and implications

Our findings illustrate that the primary bottleneck in enacting competency-based assessments is not teacher literacy but the systemic ecological constraints. However, as an exploratory single-case study, these results should be interpreted as theoretical propositions rather than universal empirical laws. While the mechanism of rational triage offers a robust explanation for this specific Grade 5 context, the findings are intended for analytic generalization—providing a conceptual framework to be tested in other educational ecologies.

For future practice and policymaking, this study offers several critical implications. First, to resolve structural constraints, school administrators must recognize assessment design and data analysis as official components of teacher workloads, or strategically adjust class sizes for inquiry-based STEM units. Second, to overcome cultural friction, the transition from a culture of correctness to a culture of evidence must be carefully scaffolded. Rather than immediately applying rubrics for formal grading, teachers should gradually integrate them into low-stakes routines (e.g., evaluating anonymous past projects) to build student trust and internalize evaluation criteria. Ultimately, the role of researcher–practitioner partnerships (RPPs) must evolve beyond merely co-designing rubrics to building sustainable implementation workflows. RPPs should actively explore technological offloading (e.g., lightweight AI tools) to alleviate the severe documentation burden, preserving educators’ energy for responsive pedagogical interventions.

## Data Availability

The datasets presented in this article are not readily available because they contain identifiable qualitative data (including classroom observation notes, interview transcripts, and students’ assessment records) involving a frontline teacher and minor students. Public sharing of this raw data would compromise participant privacy and violate the ethical confidentiality agreements. Requests to access the datasets should be directed to Fan Shi, shifanbnu@163.com.
