# Supplementary material for: Translating policy into practice: teacher agency amid cognitive and ecological constraints in enacting competency-based assessment
Source: Front Psychol. 2026 Jun 12;17:1836710. doi: 10.3389/fpsyg.2026.1836710 (PMC13308518; doi:10.3389/fpsyg.2026.1836710)
Supplement: Supplementary file 1 [file Table_1.DOCX]

**Supplementary Material**

**Article Title:** Translating Policy into Practice: Teacher Agency amid Cognitive and Ecological Constraints in Enacting Competency-Based Assessment

**Table of Contents**

**Supplementary Appendix 1:** Performance Profiles of the Four Focal Students

**Supplementary Appendix 2:** Level Descriptors for the Multidimensional Evaluative Framework

**Supplementary Appendix 3:** Student Self-assessment Scales for the ‘Making a Thermos Cup’ Unit

**Supplementary Appendix 4:** Peer-assessment Rubric for the ‘Making a Thermos Cup’ Unit

**Supplementary Appendix 5:** Teacher Assessment Portfolio

****Supplementary Appendix 1. Performance Profiles of the Four Focal Students****

| **Student** | **Representative Strengths** | **Identified Growth Areas** | **Illustrative Data Excerpt with Interpretation** |
| --- | --- | --- | --- |
| **StA** | Strong scientific interest;  Positive attitude | Foundational conceptual understanding; Model construction | "I think my design is like a dinosaur cup!"[This quote demonstrates high engagement and creativity, but also a preliminary, anthropomorphic approach to modeling, indicating an area for conceptual development.] |
| ****StB**** | Creative design ideas;  Proactive in proposing solutions | Collaboration skills;  Discipline in group work | "I don't agree with the score some peers gave me..."[This statement highlights a tension experienced during peer assessment, serving as a specific instance of the student's challenges with collaborative negotiation and accepting feedback.] |
| ****StC**** | Strong collaboration and organizational skills | Depth of conceptual application in reasoning | "After the teacher's examples, I understood how to use data to argue." [This reflects the student's responsive learning and ability to leverage scaffolding, yet it also suggests a reliance on direct instruction for applying concepts, pointing to a future growth target.] |
| ****StD**** | Systematic reasoning; Advanced model evaluation | — | "I assessed other groups' models to improve our own."[This excerpt exemplifies metacognitive awareness and high-level critical thinking, as the student strategically uses evaluation of peers' work to inform and refine their own group's model.] |

### ****Supplementary Appendix 2. Level Descriptors for the Multidimensional Evaluative Framework****

This appendix provides the detailed level descriptors for each secondary indicator within the evaluative framework. The descriptors articulate a developmental progression across four levels of proficiency (1-4), which correspond to the uni-structural to extended abstract levels of the SOLO taxonomy.

| **Primary Dimension** | **Secondary Indicator** | **Level Description** |
| --- | --- | --- |
| ****Scientific Concept**** | **Specific Scientific Concepts** | **L1:** Be Able to understand scientific concepts based on everyday experience and fragmented facts, but the understanding is vague.  **L2:** Be Able to understand the meaning of scientific concepts based on facts and experience, and can provide examples of scientific terms.  **L3:** Be Able to explain which essential characteristics of things are related to a scientific concept, and can infer qualitative or quantitative relationships between scientific terms and these characteristics based on facts and experience.  **L4:** Demonstrate a deep understanding of specific concepts, can explain the relationships between concepts and their significance, and begins to construct a systematic knowledge framework. |
|  | **Nature of Science** | **L1:** Initially perceive the nature of science, recognizing that science is a systematic inquiry into nature and that scientific knowledge is based on evidence.  **L2:** Begins to understand that scientific knowledge is subject to change and can accept that scientific theories are updated with new evidence.  **L3:** Be Able to deeply understand the constructed and tentative nature of scientific knowledge, recognizing that its development is a continuous, self-correcting process.  **L4:** Hold a profound understanding of the nature of science, can critically evaluate scientific knowledge and issues, and understands the role of science in society and culture. |
| ****Scientific Thinking**** | **Modeling** | **L1:** Can recall simple learned models but cannot use them purposefully; can compare a model to reality and judge its similarity through communication with peers.  **L2:** Can identify the function of a learned model and attempt to use it to solve problems; can identify major flaws in a model and make appropriate modifications based on authoritative information.  **L3:** Can select a suitable model to solve a problem in a given context; can evaluate the strengths and weaknesses of relevant models to choose an appropriate one; can supplement or modify a model based on empirical testing or evidence collected within a new explanatory mechanism.  **L4:** Can evaluate competing models and integrate their strengths to enhance explanatory and predictive power; can use scaffolds to reconstruct a model spontaneously based on existing elements. |
|  | **Reasoning & Argumentation** | **L1:** Can state a claim but provides no valid evidence or reasoning, failing to demonstrate a complete reasoning process.  **L2:** Support a claim by describing personal experience, prior knowledge, or specific experimental results, constituting a data-based argument.  **L3:** Analyze available data, selects valid evidence to support a claim, and explains the connection between the claim and evidence within a specific context.  **L4:** Use inductive generalization or deductive abstract relationships to support a claim, articulating more universal relationships, principles, or laws, thereby demonstrating a complete argumentation process. |
|  | **Creative Thinking** | **L1:** Propose ideas based on mindset inertia, showing little creativity.  **L2:** Engage in imagination and design based on external features of things, breaks mindset inertia for common problems, proposes somewhat novel and reasonable viewpoints, and conducts preliminary scientific analysis of solutions.  **L3:** Engage in imagination based on the structure and function of things, uses creative thinking to propose somewhat novel and reasonable viewpoints based on scientific principles; can conduct preliminary creative design and express ideas using images, text, or physical objects.  **L4:** Master and apply basic methods of creative thinking, proposes novel and reasonable viewpoints from multiple perspectives based on scientific concepts and methods, designs creative products of certain novelty and value, and demonstrates preliminary creative problem-solving ability. |
| ****Scientific Practices**** | **Scientific Inquiry** | **L1:** Be Able to conduct simple inquiry and communication based on given questions and hypotheses.  **L2:** Understand basic scientific methods (e.g., observation, experiment, reasoning, explanation), can pose inquiry questions from specific phenomena or objects, and conduct simple inquiry based on existing experience and knowledge.  **L3:** Pose inquiry questions from multiple perspectives and formulates an inquiry plan; can apply basic scientific methods to conduct complex inquiry, make observations, and keep records.  **L4:** Can pose valuable inquiry questions, design controlled experimental plans, understand the inquiry process, use various scientific methods for systematic investigation, collect and analyze experimental data, complete inquiry tasks, and write an inquiry report. |
|  | **Technological & Engineering Practice** | **L1:** Know the function and usage of simple tools; has a preliminary understanding of technical principles, production purposes, and manufacturing processes.  **L2:** Master the use of common tools; can disassemble simple products and reassemble them; can make a simplified physical model of a product that reflects some of the scientific principles involved; shows initial awareness of participating in technological and engineering practices and skills in using common tools.  **L3:** Apply learned scientific principles to successfully create simple devices with a clear process; can conduct simulation demonstrations and provide brief explanations; can improve the design and production of physical models based on evidence.  **L4:** Know the process engineering entails; can create simple devices that translate scientific principles into technological products, using tangible models to demonstrate abstract scientific principles; can simulate, analyze, and predict design schemes based on learned principles, and iteratively improve physical models based on practical feedback. |
|  | **Self-Directed Learning** | **L1:** Show weak self-directed learning ability, characterized by low willingness, passive acceptance, reliance on others' guidance and supervision, and little reflection on the learning process.  **L2:** Begin to recognize the importance of learning, can participate in the learning process, and formulate and execute learning plans under the guidance and supervision of teachers or others.  **L3:** Show willingness for active learning, can independently formulate and execute learning plans, master basic learning methods, explore learning strategies suited to personal characteristics, and monitor and adjust the learning process.  **L4:** Have established a highly autonomous learning mode, can set reasonable learning goals and plans, manage the learning process, monitor learning behaviors, reflect on learning outcomes and processes, has a clear awareness and evaluation of own learning results, and possesses a certain level of self-directed learning ability and preliminary lifelong learning awareness. |
|  | **Collaborative Learning** | **L1:** Rarely participate in group discussions, pays little attention to the group situation, expresses fragmented views, group member roles are ambiguous, the collaborative atmosphere is chaotic, and rarely helps other members.  **L2:** Participate in group discussions during problem-solving, occasionally pays attention to the group situation, expresses relatively complete views, group member roles are reasonably assigned, the collaborative atmosphere is average, and can help members in difficulty.  **L3:** Actively communicate with group members during problem-solving, consistently pays attention to the group situation, expresses views completely, group member roles are reasonably assigned, the collaborative atmosphere is positive and orderly, and actively helps members in difficulty.  **L4:** Consistently and proactively communicate with group members, pays close attention to the group situation, participates in discussions actively and seriously, expresses views completely and logically, group member roles are clear, the collaborative atmosphere is excellent and orderly, and always helps other members in difficulty. |
| ****Attitude & Responsibility**** | **Learning Interest** | **L1:** Be Interest in scientific phenomena and learning depends primarily on external factors (e.g., rewards, praise, adult expectations).  **L2:** Begin to transform external interest into preliminary internal interest. Shows curiosity about common natural phenomena or the conditions/processes of phenomena, but this interest may be unstable and easily influenced by external factors.  **L3:** Internal learning interest develop further. Shows causal interest in the processes and reasons behind phenomena, is willing to learn related knowledge, engage in theoretical study, and feels excitement and satisfaction from the inquiry process itself.  **L4:** Demonstrate a high degree of internal interest, can overcome difficulties and persevere, engages in long-term and in-depth thinking about the causes, laws, and theoretical issues of scientific phenomena, and maintains enthusiasm without external incentives. |
|  | **Scientific Attitude** | **L1:** Have little understanding of scientific knowledge and methods, tends to believe claims without scientific basis.  **L2:** Begin to contact scientific knowledge but often accepts information passively, lacking habits of active inquiry and critical thinking; may view scientific knowledge as authoritative and unquestionable, overlooking its relative and tentative nature.  **L3:** Inquire into scientific knowledge, tries to understand scientific principles and methods, is willing to ask questions, conduct experiments, seek answers, and attempts to apply scientific knowledge in real-life contexts.  **L4:** Actively inquire into scientific knowledge, dares to question, and thinks critically about scientific information; can distinguish true from false information, evaluate the reliability and value of scientific research, remains open to different viewpoints and explanations, and does not blindly follow authority. |
|  | **Social Responsibility** | **L1:** Perceive science and social development as distant and unrelated to oneself.  **L2:** Establishes a preliminary understanding of the natural world, begins to recognize the pros and cons of science for social development, and knows the importance of environmental protection and resource conservation.  **L3:** Understand the interactions between science, technology, society, and the environment (STSE), and the ethical considerations in scientific research and technological application; begins to develop an awareness of valuing life, protecting the environment, and saving resources.  **L4:** Understand STSE relationships, consciously adheres to ethical norms in scientific research and technological application, pays attention to socio-scientific issues closely related to science and technology, and develops a strong sense of responsibility for loving nature, cherishing life, saving resources, and protecting the environment. |

### ****Supplementary Appendix 3. Student Self-assessment Scales for the ‘Making a Thermos Cup’ Unit****

This appendix presents the student self-assessment scales used in the ‘Making a Thermos Cup’ unit. The scales contextualize the competencies from the evaluative framework into student-friendly language. For each item, students are asked to select the option (A, B, C, or D) that most accurately reflects their experience or perspective. The options are structured to correspond to a developmental progression across the four proficiency levels.

| **Indicators** | **Items** | **Options** |
| --- | --- | --- |
| ****Understanding the Nature of Science**** | ****Q1:** How do you understand and perceive scientific knowledge and its development process?** | ****A.**** I think scientific knowledge is solely based on evidence and cannot include scientists' personal ideas.  ****B.**** I think scientific knowledge is not always absolutely correct; it can have errors and is subject to change.  ****C.**** I think the formation of scientific knowledge isn't achieved quickly; it requires a long period of accumulation and correction.  ****D.**** I think science is part of our social and cultural traditions. Scientific knowledge and activities are influenced by society, culture, and history. |
|  | ****Q2:** How do you understand and view science and its research process?** | ****A.**** I believe scientific research is about finding the correct answer, and a scientific question usually has only one appropriate method to solve it.  ****B.**** I believe theories can be tested through experiments, and these experiments should be repeatable by other scientists with similar results.  ****C.**** I believe different research methods may suit different problems. Scientists build and revise theories through continuous experimentation and observation.  ****D.**** I believe there is no single, universal, standard procedure for scientific research. It is a complex social process, and different cultural and social backgrounds influence its development. |
| ****Model Construction**** | **Q3:** **How did you improve your thermos cup model during the making process?** | ****A.**** I compared our thermos cup model with a real one to judge their similarity.  ****B.**** I judged whether there were major defects or needs for improvement based on the heat retention effect the model was supposed to achieve.  ****C.**** I used evidence collected from the experimental process and results to supplement and modify the model, making it more refined.  ****D.**** I evaluated and compared competing models from other groups, integrating the strengths of different models to optimize and innovate our own group's design. |
| ****Reasoning & Argumentation**** | **Q4:** **How did you state the problems and conjectures regarding the differences in heat retention effects between different groups' thermos cups?** | ****A.**** I stated my findings but did not provide evidence or reasons.  ****B.**** I used the test data from each group's thermos cup model to explain my findings.  ****C.**** I combined test data and explained my findings and conjectures from the perspective of how materials affect heat retention.  ****D.**** Based on the different materials and insulation methods used by each group, I summarized the relationship between material thermal conductivity, blocking heat transfer, and the heat retention effect, and was able to present a complete argument. |
| ****Creative Thinking**** | ****Q5:** What kind of ideas did you contribute during the formulation, improvement, and upgrading of your group's thermos cup design?** | ****A.**** The ideas or suggestions I proposed were the same as others'.  ****B.**** I proposed my own ideas regarding the appearance of the thermos cup during its design, improvement, and upgrading.  ****C.**** I proposed my own ideas regarding the structure and function of the thermos cup during its design, improvement, and upgrading.  ****D.**** During design, improvement, and upgrading, I proposed novel ideas from multiple perspectives based on the methods of heat transfer and the principles of thermal insulation. |
| ****Self-Directed Learning Ability**** | ****Q6:** How did you manage your learning goals and behaviors in the 'Making a Thermos Cup' activity?** | ****A.**** I had difficulty managing my learning behaviors, participated little in class activities, and needed supervision from the teacher or classmates.  ****B.**** I consciously participated in class activities and completed the set learning tasks with the help of the teacher and other students.  ****C.**** I actively participated in class activities, monitored my own progress during learning, and strived to keep up with the group.  ****D.**** I made a learning plan, managed the learning process, proactively collected relevant information after class to complete the tasks, and was able to reflect on the learning outcomes and process. |
| ****Scientific Inquiry Ability**** | ****Q7:** How did you design the investigation into the factors affecting the heat retention performance of the thermos cup?** | ****A.**** I was able to conduct simple inquiry and communication based on the given questions and hypotheses.  ****B.**** I was able to investigate the impact of materials on heat retention by combining life experience and knowledge about material thermal conductivity.  ****C.**** I was able to formulate an inquiry plan based on the question, use experimental and observational methods to investigate the factors affecting heat retention, and record the experimental data.  ****D.**** I was able to design a controlled experiment to investigate the question, collect and analyze data to verify the hypothesis. |
| ****Technological & Engineering Practice Ability**** | ****Q8:** How did you perform during the process of making the thermos cup model? What was the outcome?** | ****A.**** I know the function and use of a thermos cup, but I could not use the available materials to make a finished product.  ****B.**** I did not make a thermos cup model independently, but I could disassemble and reassemble the one made by my group.  ****C.**** I successfully made a thermos cup model, could demonstrate its heat retention, and briefly explain its principle.  ****D.**** I successfully made a thermos cup model, could predict and analyze its heat retention performance, iteratively improved the model based on actual test results, and explained the abstract principle of blocking heat transfer for insulation. |
| ****Learning Interest**** | ****Q9:** Do you think the 'Making a Thermos Cup' activity was interesting? Why?** | ****A.**** I found this activity boring and participated only because it was a task assigned by the teacher.  ****B.**** I found the hands-on making part of the activity interesting. I enjoyed the process and wanted to make a cup with good heat retention.  ****C.**** I found the activity interesting because, by investigating the factors affecting heat retention and finding methods to block heat transfer, we successfully made a thermos cup model.  ****D.**** I found the activity very interesting because it deepened my understanding and application of knowledge related to heat. |
| ****Scientific Attitude**** | ****Q10:** What is your view of your group's thermos cup model and its test data?** | ****A.**** Our group's thermos cup model looks perfect on the surface, so the test data must be ideal.  ****B.**** Our group's thermos cup model used materials with poor thermal conductivity learned from the textbook, so the test data must be ideal.  ****C.**** Our group's thermos cup model has aspects that need improvement; the test data is an honest record of its heat retention performance.  ****D.**** Our group's thermos cup model has both strengths and aspects needing improvement. We need to learn from excellent designs based on the test data for innovation and improvement. |
|  | ****Q11:** How do you view the comments and suggestions others gave about your group's thermos cup presentation?** | ****A.**** I accepted the positive parts of the comments and suggestions but resisted or refuted the negative parts.  ****B.**** I accepted the comments and suggestions but couldn't truly understand and digest them, so I didn't actually incorporate the suggestions into my thinking and actions.  ****C.**** I held an open and inclusive attitude towards others' comments and suggestions, actively listened to them, and thought deeply about their substance and meaning.  ****D.**** I could think critically about the comments and suggestions, used the beneficial advice for our own purposes, and sought further feedback and suggestions after making improvements. |
| ****Social Responsibility**** | ****Q12:** Through learning about heat preservation and dissipation technologies, how do you understand the relationship between technology and society/environment?** | ****A.**** The relationship between thermal technology and society/environment seems distant to me; my understanding is superficial.  ****B.**** I think technologies like thermal insulation bring convenience to social life, and technology does not have negative impacts on society and the environment.  ****C.**** I think technology is a double-edged sword for society and the environment. We should actively take actions to reduce negative impacts, such as using more environmentally friendly insulation materials and optimizing cooling systems to reduce energy consumption.  ****D.**** I believe technology, society, and the environment are closely related. We should internalize the fulfillment of social responsibility as our behavioral norm, prioritize environmental factors during production and use, and consciously adopt responsible behaviors to save resources and protect the environment. |

### ****Supplementary Appendix 4. Peer-assessment Rubric for the ‘Making a Thermos Cup’ Unit****

****Team Name:**** ____________________ ****Evaluator (Your Name):**** ____________________

****Instructions:**** Please rate the performance of your team members in the table below using a scale of 1 to 4. The score should most accurately reflect the actual situation of the team members.

****(1 — Needs Improvement;2 — Satisfactory;3 — Good;4 — Excellent)****

| **Evaluation Dimension** | **Evaluation Criteria** | **Team Member** | | | | |
| --- | --- | --- | --- | --- | --- | --- |
|  |  | **1** | **2** | **3** | **4** | **5** |
| ****Collaborative Learning Ability**** | 1. Be able to clarify their own role and responsibilities and was willing to accept assigned tasks. |  |  |  |  |  |
|  | 2. Be able to cooperate actively with other team members and participate in group discussions. |  |  |  |  |  |
|  | 3. Contributed positively to the formation of a good and orderly collaborative atmosphere within the group. |  |  |  |  |  |
| ****Scientific Inquiry Ability**** | 4. Be able to put forward one's own hypotheses and conjectures regarding the significant differences in the heat preservation effects of each group of thermos cups |  |  |  |  |  |
|  | 5. Actively participated in designing controlled experimental plans and took part in the inquiry activities. |  |  |  |  |  |
|  | 6. Be able to communicate and analyze experimental data with team members to investigate influencing factors and draw conclusions. |  |  |  |  |  |
| ****Technological & Engineering Practice Ability**** | 7. Participated in the initial construction and improvement of the thermos cup device, and was able to collaborate in making the "thermos cup" device based on scientific principles of heat. |  |  |  |  |  |
|  | 8. Actively participated in simulation demonstrations and was able to briefly explain the testing process and results, describing its working principles. |  |  |  |  |  |
| ****Total Score**** |  |  |  |  |  |  |

****Additional Comments (Please record specific instances and details):****
__________________________________________________________________________________________________________________________________________________________________________________________________________________

_________________________________________________________________________________________________________

_________________________________________________________________________________________________________

### ****Supplementary Appendix 5: Teacher Assessment Portfolio****

#### ****Part A: Dual-Function Narrative Log (Competencies & Implementation)****

****1. Purpose and Scope****

This Narrative Log serves a dual purpose:

(a), Assessment of Learning: To document observable evidence of student competencies using the structured indicators (see Exemplar Table A5-1).

(b), Implementation Reflection: To record holistic observations of instructional barriers (e.g., timing issues, management load) in the narrative notes following each session.

****When to Record:****

*For Student Competencies:* Make entries during or immediately after key learning activities (e.g., group design sessions, presentation of arguments).

*For Implementation Reflection:* Make brief entries post-lesson to capture the holistic reality of enactment without disrupting instruction.

****Focus of Entries:****

*Student Data:* Record concrete and specific evidence (direct quotes, descriptions of actions, sketches of models) rather than general impressions.

*Reflection Data:* Record specific structural constraints (e.g., “activity X took 10 mins longer than planned”) or frictions (e.g., “students struggled to use the peer rubric”).

****Linking to Framework** (For Student Entries Only)**:**** For entries regarding student performance, note the relevant **Secondary Indicator** and propose a **Provisional Proficiency Level (1-4)** based on the framework descriptors.

****2. Exemplar Log Entries****

The following table provides anonymized examples from the implementation of the ‘Making a Thermos Cup’ unit, illustrating how observations were documented and preliminarily analyzed.

****Table A5-1: Exemplar Student Assessment Entries****

| **St** | **Observation**  **(Direct Quote / Description of Action)** | **Relevant Indicator** | **Provisional Level** | **Analytical Note**  **(Rationale)** |
| --- | --- | --- | --- | --- |
| **StA** | Holding up cup: "Look! It's a dinosaur. This is the head, this is the tail."  (Focused entirely on shape, not heat) | ****Modeling**** | L2 | **Analogy-based.** Uses simple metaphor (dinosaur) to represent structure. Focus is aesthetic, not functional/insulative. |
| **StC** | After checking Group B's data: "Wait, theirs stayed hot longer... I saw they used a lid and layered cotton. We only have one layer. Let's add a lid?" | ****Reasoning & Arg.**** | L3 | **Data-Evidence Link.** Connects others' results to specific design features. Proposes valid fix. |
| **StB** | Sketching double-walls: "If air is a good insulator... maybe trapping it between two walls is better than just wrapping it?" | ****Creative Thinking**** | L3 | **Concept Transfer.** Applies abstract principle to a novel structural design (double-wall). Divergent thinking. |

****Part B: Energy Concept Surveys****

一、选择题（每个问题有且仅有一个正确答案）

1. 夏天的电风扇带来凉风。下列关于能量形式的说法，正确的是（）
2. 电风扇工作时，消耗电能
3. 电风扇工作时，产生电能
4. 电风扇工作时，不产生动能
5. 如图所示，一个滑板运动员站在U字形滑道的顶端。下列说法正确的是（）
6. 此时他还没有出发，不具有动能
7. 当他第一次到达滑道底端时，不具有动能
8. 当他停留在滑道底端时，不具有动能


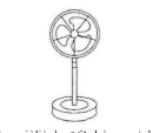

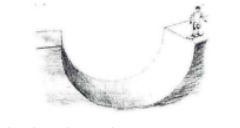


第1题 第2题

二、判断题（正确打“√”，错误打“×”）

1. 如图所示，火力发电厂是靠可燃物（例如煤）燃烧发电。从能量转化情况来看，煤的化学能最终转化成了电能。（）
2. 如图所示，小球被拉高一段距离，开始摆动。小球和周围环境（包括空气、绳子等）的总能量是守恒的。（）
3. 如图所示，旋转的风车能够发电。风车旋转时的动能大于产生的电能。是因为能量利用率没有达到100%。（）


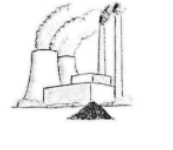

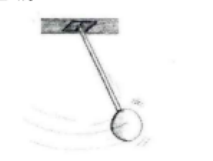

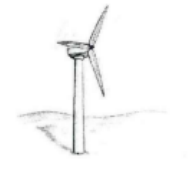


第3题 第4题 第5题

三、填空题

1.
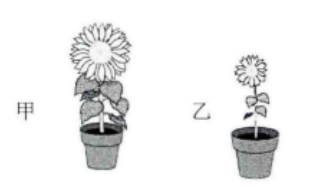
甲和乙各有一棵来自同一向日葵的种子，在同一天把它们种进相同的花盆和土壤里，经过几个月的照料，他们的向日葵如图所示。下列说法合理的是_____________（只要填出选项）
2. 向日葵的生长和能量有关
3. 甲的向日葵吸收了更多能量
4. 乙的向日葵吸收了更多能量
5. 为了让向日葵长得像甲一样，乙需 要浇更多的水和晒更多的太阳
6. （1）下图中，_____冰块融化最慢，________冰块融化最快（只要填出选项）。

（2）从“能量守恒”解释原因

_______________________________________________________________


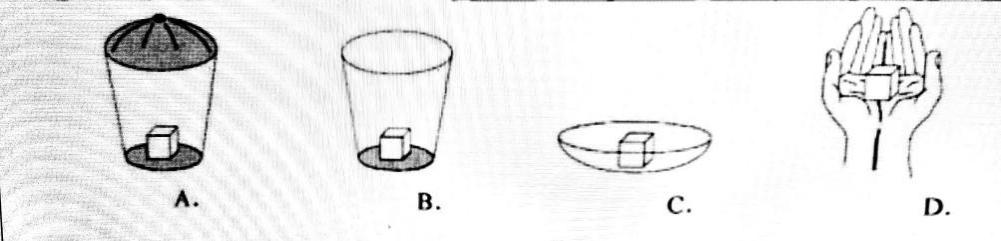


1. 下列选项属于能源的是：_________________________________（只要填出选项）
2. 煤 B.石油 C.天然气 D.太阳能 E.风能 F.水能 G.电能 H.化学能
3. 下列常见器材或设备的工作原理是将什么能转化成什么能？
4. 电饭煲：_____________________
5. 太阳能电池：________________________
6. 核能发电：________________________
7. 电灯：_________________________
